# Mechanisms of primordial follicle activation and new pregnancy opportunity for premature ovarian failure patients

**DOI:** 10.3389/fphys.2023.1113684

**Published:** 2023-02-28

**Authors:** Tuo Zhang, Meina He, Jingjing Zhang, Yuntong Tong, Tengxiang Chen, Chao Wang, Wei Pan, Ziwen Xiao

**Affiliations:** ^1^ Department of Obstetrics and Gynecology, The Affiliated Hospital of Guizhou Medical University, Guiyang, China; ^2^ Transformation Engineering Research Center of Chronic Disease Diagnosis and Treatment, Department of Physiology, College of Basic Medicine, Guizhou Medical University, Guiyang, Guizhou, China; ^3^ Prenatal Diagnosis Center in Guizhou Province, The Affiliated Hospital of Guizhou Medical University, Guiyang, China; ^4^ College of Basic Medicine, Guizhou Medical University, Guiyang, Guizhou, China; ^5^ Guizhou Provincial Key Laboratory of Pathogenesis and Drug Research on Common Chronic Diseases, Department of Pathophysiology, School of Basic Medical Sciences, Guizhou Medical University, Guiyang, Guizhou, China; ^6^ Guizhou Institute of Precision Medicine, Affiliated Hospital of Guizhou Medical University, Guiyang, China; ^7^ State Key Laboratory of Agrobiotechnology, College of Biological Sciences, China Agricultural University, Beijing, China

**Keywords:** ovary, follicle, primordial follicle activation, *In vitro* activation, premature ovarian failure

## Abstract

Primordial follicles are the starting point of follicular development and the basic functional unit of female reproduction. Primordial follicles are formed around birth, and most of the primordial follicles then enter a dormant state. Since primordial follicles are limited in number and can’t be renewed, dormant primordial follicles cannot be reversed once they enter the growing state. Thus, the orderly occurrence of primordial follicles selective activation directly affects the rate of follicle consumption and thus determines the length of female reproductive lifespan. Studies have found that appropriately inhibiting the activation rate of primordial follicles can effectively slow down the rate of follicle consumption, maintain fertility and delay ovarian aging. Based on the known mechanisms of primordial follicle activation, primordial follicle *in vitro* activation (IVA) technique has been clinically developed. IVA can help patients with premature ovarian failure, middle-aged infertile women, or infertile women due to gynecological surgery treatment to solve infertility problems. The study of the mechanism of selective activation of primordial follicles can contribute to the development of more efficient and safe IVA techniques. In this paper, recent mechanisms of primordial follicle activation and its clinical application are reviewed.

## Introduction

**FIGURE 1:**
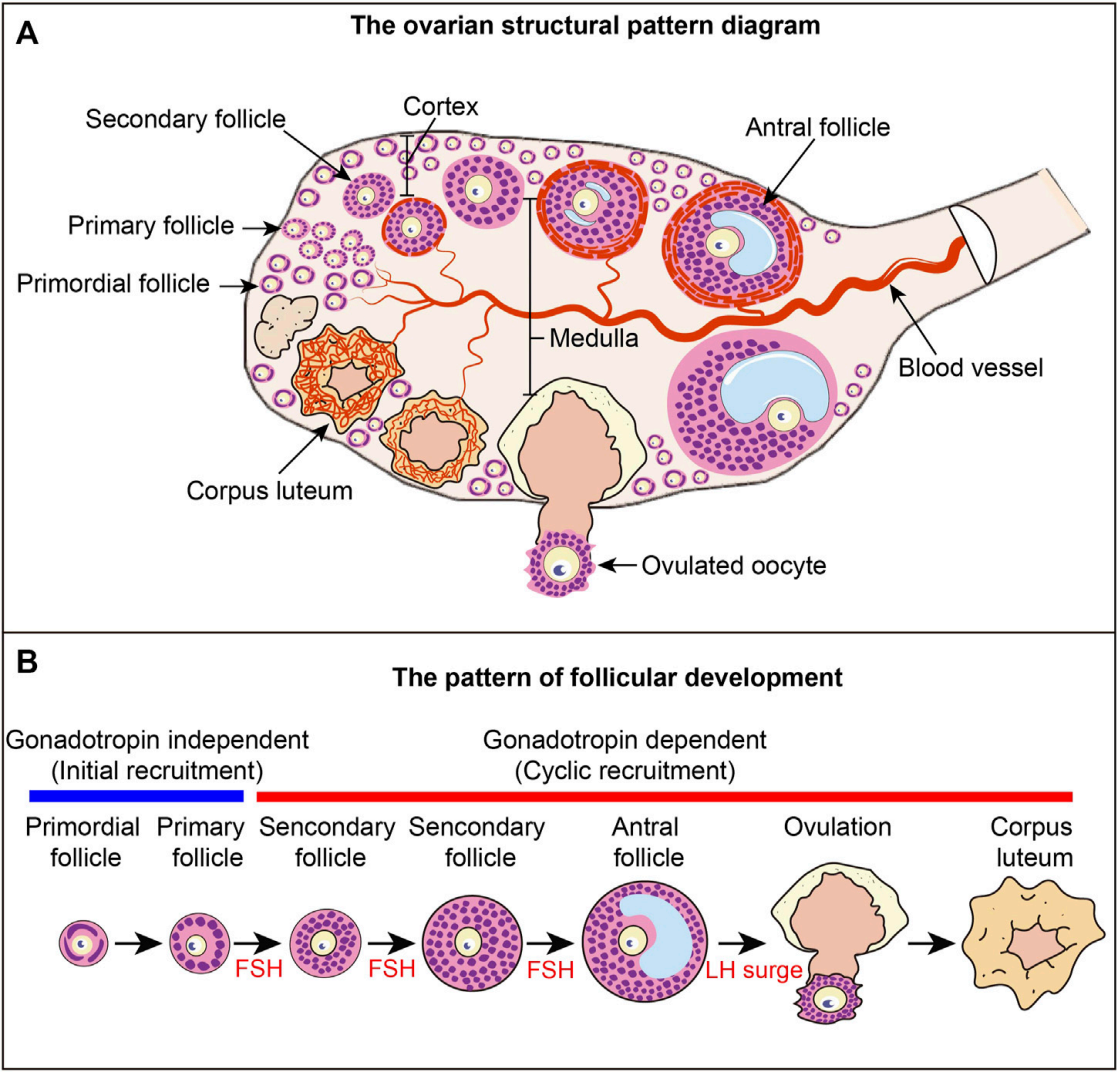
Ovarian structure and follicular development pattern diagram **(A)** Ovarian structural pattern diagram. Follicles at different developmental stages constitute the basic structural and functional unit of the ovary. Primordial follicles are mainly distributed in the cortex, and growing follicles are mainly distributed in the medulla. The blood vessels in the ovary provide nutrients to the ovarian tissue and follicles, and the blood vessels around each follicle are relatively independent. **(B)** Follicular development pattern. The development of primordial follicles into primary follicles is independent of gonadotropins, a process called primordial follicle activation, also known as initial recruitment. The process from primary follicle development to ovulation is a gonadotropin-dependent stage. The process of primary follicle development into preantral follicles is called cyclic recruitment and is the FSH response phase. Antral follicles ovulate under the stimulation of the LH surge. After ovulation, the granulosa cells luteinize to form the corpus luteum.

**FIGURE 2:**
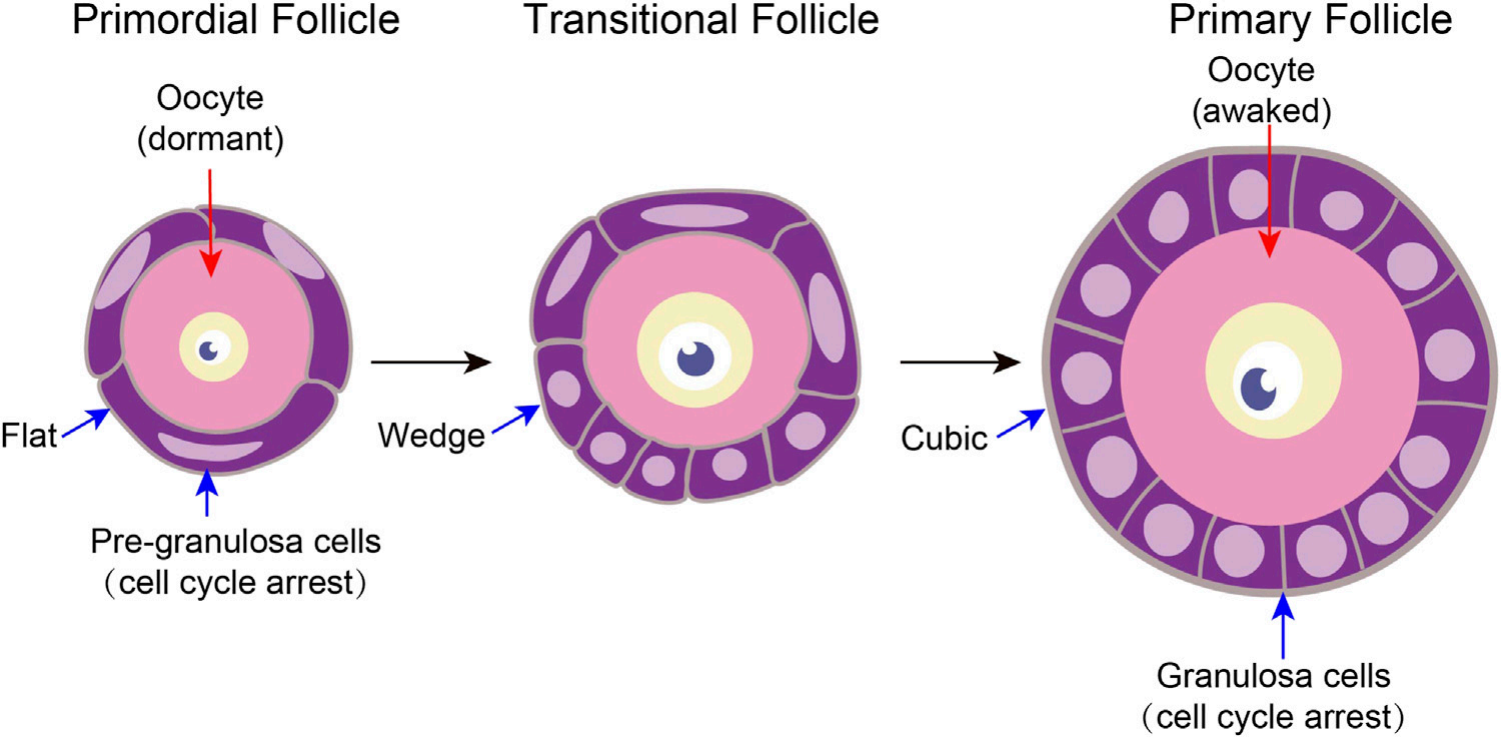
Characteristics of tissue structure changes in primordial follicle activation A follicle consists of a single oocyte in the middle and several somatic cells surrounding it. Primitive follicle activation mainly has two characteristics of structural changes. On the one hand, after primordial follicle activation, the pre-granulosa cells slowly changed from flat to wedge-shaped, and then to cubic-shaped. On the other hand, the oocyte diameter increased.

Ovary is an important reproductive and endocrine organ for female mammal. The normal ovarian function provides a fundamental guarantee for the body’s suitable reproductive life and stable endocrine environment. There are two types of follicles in the adult ovarian follicle pool, one is the growing follicle, and the other is large number of primordial follicles as ovarian reserve. The primordial follicle pool is not renewable, and the primordial follicle cannot be reversed once it enters the growing state (Zhang et al., 2015; Zhang and Liu, 2015; Kallen et al., 2018). Therefore, the orderly primordial follicle activation plays a decisive role in maintaining the length of female reproductive life (Reddy et al., 2010; Zhao et al., 2021). There are different stages during follicles development included primordial follicle, primary follicle, secondary follicle, antral follicle and preovulatory follicles in the ovary, but most of the follicles are primordial follicles, and these primordial follicles are in a dormant and static state (Pedersen, 1970; Hsueh et al., 2015; Zhang et al., 2015; Monget et al., 2021) (Figure 1). Primordial follicles consist of a single central oocyte surrounded by multiple pre-granulosa cells. Interestingly, the oocytes are arrest in the first meiosis stage and their growth is relatively static, the cell cycle of the pre-granulosa cells is inhibited (Jaffe and Egbert, 2017; Granados-Aparici et al., 2019). This state can be maintained as long as a year in mice, and up to 50 years in humans. These dormant primordial follicles are recruited from the primordial follicle pool and enter the growth follicle stage. This process is named the initial recruitment, also called primordial follicle activation (Lintern-Moore and Moore, 1979). Primordial follicle initial recruitment is different from cyclic recruitment (Kallen et al., 2018). It is generally believed that cyclic recruitment is regulated by gonadotropins, while initial recruitment is not regulated by gonadotropins (McGee and Hsueh, 2000; Bian et al., 2021). Primordial follicle initial recruitment is mainly regulated by the signals in the pre-granulosa cells and oocyte, as well as by conditions such as growth factors and stress in the primordial follicle microenvironment (Bian et al., 2021). After the primordial follicle is activated, the pre-granulosa cells gradually change from flat to wedge-shaped, then cuboidal, and later called granulosa cells. Meanwhile, oocyte diameter increased (Kallen et al., 2018) (Figure 2). When the primordial follicle is activated to enter the growth follicle stage, it enters the irreversible growth and development process, so the activation of the primordial follicle is equivalent to a gate for follicular development (Hirshfield, 1991; Adhikari et al., 2009). In order to maintain a suitable length of reproductive life and the reproductive health of the body, primordial follicles in the ovaries need to be properly activated at the right time (Zhang and Liu, 2015; Chen et al., 2022). The understanding of the mechanisms of primordial follicle activation is still limited. To better grasp the progress of research on primordial follicle activation, we summarize the currently known key networks that regulate the activation of primordial follicles in this review.

## Two waves of primordial follicles activation in mouse

Primordial follicles are formed around birth. The primordial follicles in the ovarian medulla are synchronously activated to become the first wave of activated follicles. The dormant primordial follicles in the cortical region of the ovary are gradually activated into a second wave of activated follicles (Hirshfield, 1991; Mork et al., 2012). Primordial follicles are mainly stored in the cortical area, and growing follicles are mainly stored in the medullary area. The developmental dynamics, functions and mechanisms of the two waves of follicles are different (Zheng et al., 2014; Dai et al., 2022). The activation of the first wave primordial follicles in the medulla is regulated by oocytes and has been determined during the formation of primordial follicles in the embryonic stage (Dai et al., 2022). However, the activation of the second wave primordial follicles in the medulla of the adult ovary may be regulated by pre-granulosa cells (Zhang et al., 2014). The first wave of primordial follicle development later contributes to the onset of puberty. The second wave of primordial follicles contributes to the entire reproductive process in adulthood (Zheng et al., 2014). The current understanding of the mechanism of the two waves of primordial follicle activation is limited, and this will be a fundamental scientific topic that needs attention in the development of primordial follicles.

## The signal pathway in oocyte

### PTEN-PI3K-AKT signaling

Primordial follicles are composed of only two types of cells: oocytes and pre-granulosa cells. The activation of primordial follicles requires the participation of these two types of cells (Zhang et al., 2015). In the process of primordial follicle activation, two signaling pathways, the phosphatidylinositol-3 kinase (PI3K) signaling pathway and the mechanistic target of rapamycin complex (mechanistic target of rapamycin complex 1, mTORC1) signaling pathway play a key role. PI3K signaling in oocytes is required for primordial follicles to maintain dormant state and follicular reserve (Adhikari and Liu, 2009; Adhikari and Liu, 2010; Zhang et al., 2014; Maidarti et al., 2020; Zhao et al., 2021). Phosphatase and tensin homolog (PTEN) negatively regulate intracellular levels of phosphatidylinositol-3,4,5-trisphosphate (PIP3) in cells and functions as a tumor suppressor by negatively regulating protein kinase B (PKB/AKT) signaling pathway (Worby and Dixon, 2014; Yehia et al., 2020). PTEN-PI3K-AKT is a relatively well-studied and clear signaling pathway during primordial follicle activation (Reddy et al., 2005; Liu et al., 2006). PTEN is mainly localized in dormant primordial follicle oocytes, deletion of *Pten* in primordial follicle oocytes will lead to excessive activation of the PI3K signaling pathway in oocytes, leading to premature activation of primordial follicles and ultimately premature ovarian failure (Reddy et al., 2008). Pyruvate dehydrogenase kinase 1 (PDK1) activates AKT through co-binding to PIP3 generated by PI3Ks (Gagliardi et al., 2018). Conditional knockout of *Pdk1* in primordial follicle oocytes results that the majority of primordial follicles are depleted around the onset of sexual maturity. PTEN-PDK1 signaling in oocytes that controls the survival, loss and activation of primordial follicles (Reddy et al., 2009).

### TSC1/TSC2-mTOR signaling in oocyte

mTOR is essential for oogenesis, follicular development, maintenance of follicular reserve, and oocyte maturation (Liu et al., 2018; Correia et al., 2020). TSC complex subunit 1 (TSC1) and TSC complex subunit 2 (TSC2) negatively regulates mammalian target of rapamycin complex 1 (mTORC1) signaling. mTOR is a conserved kinase that mediate cellular responses to stresses such as nutrient deprivation, growth factors and DNA damage (Salussolia et al., 2019). Deletion of *Tsc1/2* in primordial follicle oocytes will lead to overactivation of the mTOR signaling pathway in oocytes, which will also lead to the premature activation of primordial follicles and eventually lead to premature ovarian failure (Adhikari et al., 2009). However, primordial follicles are normally activated in the absence of mTOR in primordial follicle oocytes, but subsequent follicle development is arrested, and granulosa cells transform into sertoli-like cells (Guo et al., 2018).

### LHX8

LIM homeobox 8 (LHX8) is a member of the LIM homeobox family of proteins (Hobert and Westphal, 2000). In the ovary, LHX8 is specifically expressed in the oocyte nucleus and involved in oogenesis, oocytes differentiation, primordial follicle activation (Choi et al., 2008; Zhao et al., 2022). *Lhx8*-null mice had abnormally increased level of autophagy in oocytes and increased oocyte DNA damage, resulting in massive oocyte loss (Ren et al., 2015; D’Ignazio et al., 2018). Wang’s study found that the oocyte-specific transcription factors LHX8, FIGLA and SOHLH1 form a transcriptional regulatory network to regulate oogenesis (Wang et al., 2020b). LHX8 directly regulates *Lin28a* transcription in primordial follicle oocytes to regulate postnatal folliculogenesis (Ren et al., 2015; D’Ignazio et al., 2018).

### CDC42

Cell Division Cycle 42 (CDC42) is a small GTPase of the Rho-subfamily, which regulates signaling pathways that control diverse cellular functions including cell morphology, migration, endocytosis and cell cycle progression (Heinrich et al., 2021; Campbell et al., 2022; Wirth et al., 2022). The subcellular localization of CDC42 during primordial follicle activation is interesting. In dormant primordial follicles, CDC42 is specifically expressed in the oocyte cytoplasm. When primordial follicles are activated, the expression of CDC42 on the oocyte membrane is greatly enhanced, the expression of the GTP-active form of CDC42 is enhanced on the oocyte membrane. CDC42 binds to P110-β protein, regulates the activation of PI3K signaling pathway in oocytes, and promotes primordial follicle activation (Yan et al., 2018). In Yan’s study, it was found that the expressions of CDC42 and PTEN in primordial follicle oocytes are mutually exclusive, but the specific regulatory relationship between CDC42 and PTEN is not clear, which needs to be explored in future research.

### E-cadherin

Cell adhesion is essential for tissue structure and function. The cadherin family members play a key role in cell-cell recognition and adhesion and interact with intracytoplasmic proteins through adaptor proteins (Collins et al., 2017). E-cadherin, a classical cadherin of the cadherin superfamily, is a calcium-dependent cell adhesion molecule that is involved in the establishment and maintenance of epithelial cell morphology during embryogenesis and adulthood (Zaidel-Bar, 2013). E-cadherin is specifically localized to the cytomembrane of oocytes in primordial follicle. E-cadherin in primordial follicle oocytes plays an indispensable role in the maintenance of the primordial follicle pool by facilitating follicular structural stability and regulating NOBOX expression (Yan et al., 2019). The study also demonstrates that oocyte-derived factors are necessary for the maintenance of follicles.

## The signal pathway in pre-granulosa cells

### TSC1/TSC2-mTOR signaling in pre-granulosa cells

Interestingly, primordial follicles failed to be activated after deletion of *Rptor*, a key member of the mTORC1 complex in pre-granulosa cells, and primordial follicles were hyperactivated after deletion of TSC1/2 (Zhang et al., 2014). Studies using multiple transgenic mouse models reveal that pre-granulosa cells initiate and govern the activation of the second wave of primordial follicles (Zhang et al., 2014; Zhang et al., 2015). Under the stimulation of surrounding factors such as hypoxia, nutritional factors, stress and other factors, mTOR in pre-granulosa cells is upregulated, pre-granulosa cells grow and differentiate into granulosa cells, and they secrete more KIT ligands at the same time (Zhang et al., 2014). KIT ligands bind to KIT receptors on the oocyte membrane and activate the PI3K signaling pathway in the oocyte (Kissel et al., 2000; Nilsson and Skinner, 2004; Hutt et al., 2006; Zhang et al., 2014; Saatcioglu et al., 2016). This enables downstream FOXO3A to be phosphorylated, and FOXO3a is transported out of the nucleus to relieve the inhibition of oocyte growth, thereby enabling primordial follicle activation (Castrillon et al., 2003; Zhang et al., 2014; Ezzati et al., 2015). Other studies have also found that CREB, MAPK, HDAC6, NGF and other molecules can regulate the activation of primordial follicles through the mTOR signaling pathway (He et al., 2017; Zhao et al., 2018; Li et al., 2020; Zhang et al., 2021b; Zhang et al., 2022). These studies further illustrate the important role of the mTOR signaling pathway in the activation of primordial follicles.

### FOXL2

Foxl2 forkhead box L2 (FOXL2), a forkhead transcription factor, contains a fork-head DNA-binding domain and it may play a role in ovarian development and function (Benayoun et al., 2011; Georges et al., 2013). Expansion of a polyalanine repeat region and other mutations in FOXL2 are a cause of blepharophimosis syndrome, premature ovarian failure and granulosa cell tumour (De Baere et al., 2002; Nallathambi et al., 2007; Pierini et al., 2020). The formation of primordial follicles in *Foxl2* knockout mice was not affected, but the pre-granulosa cells failed to differentiate and remained flat, resulting in no growing follicles in the ovary and female mice were sterile (Schmidt et al., 2004). This study also demonstrates that the developmental status of pre-granulosa cells is critical for the activation of primordial follicles and the development of subsequent growing follicles.

### SMAD3

SMAD family member 3 (SMAD3) is known to serve as a signaling intermediate for the transforming growth factor beta TGF family (Kawabata et al., 1998) *Smad3* knockout mice are viable. Notably, primordial follicle formation was not affected in *Smad3* knockout mice but delayed the activation of primordial follicles and development of growing follicles, resulted in reduced fertility (Tomic et al., 2002). The transcription factor Smad3 is expressed in the nucleus of pre-granulosa cells. SMAD3 directly regulates the transcription of CCND2 and inhibits the expression of Myc. CCND2 is bound by p27, thereby arresting the cycle of precursor granulosa cells and maintaining the dormant state of primordial follicles. When the level of TGF-β increases, SMAD3 is transported out of the nucleus, p27 dissociates from CCND2 to relieve the inhibition of the pre-granulosa cell cycle and promote the activation of primordial follicles (Granados-Aparici et al., 2019). From the current research, p27 and SMAD3 play key roles in follicle development and oogenesis, and they may regulate primordial follicle activation mainly by affecting the pre-granulosa cell cycle (Rajareddy et al., 2007). However, *p27* and *Smad3* knockout mice were used in the current study, but the precise roles and mechanisms of p27 and SMAD3 in primordial follicle activation cannot be fully elucidated, so further research is needed.

### AMH

Anti-mullerian hormone (AMH) is a secreted ligand of the TGF-β superfamily (Pepinsky et al., 1988; Howard et al., 2022). AMH is exclusively produced by granulosa cells of ovarian follicles during the early stages of follicle development (Moolhuijsen and Visser, 2020). AMH plasma levels reflect the continuous non-cyclic growth of small follicles, thereby mirroring the size of the resting primordial follicle pool and thus acting as a useful marker of ovarian reserve (Dewailly et al., 2014). AMH is the best measure of ovarian reserve in different clinical conditions at present. (Teede et al., 2019; Shrikhande et al., 2020; Vatansever et al., 2020). AMH supplementation is able to maintain follicular reserve in some ovarian injury models, such as chemotherapy-induced premature ovarian failure, polycystic ovary syndrome (PCOS) (Sonigo et al., 2019; Hoyos et al., 2020; Ou et al., 2021; Rudnicka et al., 2021).

### ESR2

Estrogen and its receptors play an integral role in the periodic recruitment of growing follicles, and estrogen receptor knockout mice lead to infertility in female mice due to abnormal meiosis (Shoham and Schachter, 1996; Liu et al., 2017; Tang et al., 2019). A recent study found that disruption of estrogen receptor β (ESR2) signaling results in increased protein level of AKT and mTOR in both granulosa and oocyte factors and leading to increased activation of primordial follicles (Chakravarthi et al., 2020). This study suggests that estrogen receptors may have no effect on the activation of the first wave of primordial follicles but may regulate the activation of the second wave of primordial follicles. It is also possible that the deletion of the estrogen receptor leads to the development of growth follicles, and the changes in the ovarian microenvironment lead to abnormal activation and loss of primordial follicles.

### Wnt ligand secretion mediator

Wingless-type MMTV integration site family (WNT) signaling is an evolutionarily conserved system for cell-cell communication (Liu et al., 2022; Rim et al., 2022). WNT is classified broadly into canonical β-catenin dependent, and non-canonical β-catenin-independent pathways (Clevers and Nusse, 2012; Nusse and Clevers, 2017). The WNT signaling pathway is indispensable for primordial germ cell development, oogenesis, follicle development, and maintenance of follicular reserve (Kocer et al., 2008; Zhou et al., 2012; De Cian et al., 2020; Habara et al., 2021). Among the 19 WNT ligands, the mRNAs for *Wnt4*, *Wnt6* and *Wnt11* were experssed in the pre-granulosa cells during primordial follicle activation. *Wnt2*, *Wnt2b*, *Wnt9a*, *Wnt5b*, *Wnt11* and *Wnt16* was expressed in the oocytes of primordial follicles (Habara et al., 2021). After conditional knockdown of the wntless in pre-granulosa cells, the pre-granulosa cells could not grow and differentiate into cubic granulosa cells, which leads to female infertility (Habara et al., 2021). Wntless in pre-granulosa cells is essential for communication between pre-granulosa cells and oocytes and primordial follicle activation.

## Other important molecules and relative signaling pathways

### HIPPO

The HIPPO pathway was first discovered in *Drosophila melanogaster*, the pathway name comes from the fact that *Drosophila* overgrew like a hippopotamus after mutations in key molecules of the HIPPO pathway in the head and eyes of *Drosophila*. The HIPPO pathway is highly conserved from *Drosophila* to mammals (Ma et al., 2019; Moya and Halder, 2019). The upstream membrane protein receptors of the Hippo signaling pathway act as receptors for extracellular growth inhibition signals, and once they sense the extracellular growth inhibition signals, they activate a series of kinase cascade phosphorylation reactions that eventually phosphorylate the downstream effectors Yes-associated protein (YAP) and transcriptional coactivator with PDZ-binding motif (TAZ). And cytoskeletal proteins bind to the phosphorylated YAP and TAZ, causing them to remain in the cytoplasm and reduce its cytosolic activity, thus achieving the regulation of regulation of organ size and volume (Huang et al., 2005; Yu et al., 2015; Ma et al., 2019). After ovarian fragmentation promotes actin polymerization, p-YAP levels decrease and promotes nuclear transfer of YAP. Nuclear localization of YAP further promotes the expression of CCN growth factors and BIRC apoptosis inhibitors, which ultimately promotes follicular overgrowth (Li et al., 2010; Kawamura et al., 2013). The changes in the HIPPO pathway after ovarian fragmentation are a double-edged sword, on the one hand they can lead to follicular overgrowth and premature ovarian failure due to early follicular depletion. On the other hand, this property can be used to promote primordial follicle activation and develop primordial follicle *in vitro* activation techniques to help infertile patients to conceive children.

### HDAC6

Histone deacetylase 6 (HDAC6) is a special histone deacetylase with two deacetylation domains and one ubiquitination domain. HDAC6 plays a center role in several processes, including positive regulation of peptidyl-serine phosphorylation, protein deacetylation, protein destabilization, microtubule stability (Olzmann et al., 2007; Wang et al., 2018; Wang et al., 2020a; Osseni et al., 2020; Wang et al., 2022). Our study showed that histone deacetylase HDAC6 was expressed heterogeneously in different primordial follicles. About 3%–4% of primordial follicles in neonatal and adult mouse ovaries had low HDAC6 expression, and 65% of primordial follicles with low HDAC6 expression will be activated. Further studies found that HDAC6 was transiently downregulated during primordial follicle activation, mediating selective activation of mouse primordial follicles by regulating the expression of mTOR (Zhang et al., 2021b). Interestingly, overexpression of *Hdac6* extends fecundity in female mice, longer telomeres and reduced DNA damage may reduce tumorigenesis in *Hdac6* overexpression mice (Zhang et al., 2017). Combined with these studies, we speculate that HDCA6 may regulate primordial follicles to selectively activate primordial follicles, prolong follicular cell telomere length and reduce DNA damage, and ultimately prolong female reproductive lifespan.

### SIRT1

NAD-dependent protein deacetylase Sirtuin-1 (SIRT1) has been reported to be involved in the regulation of cellular senescence, aging and organism longevity through the acetylation and deacetylation of these substrates altering their transcriptional and enzymatic activities, as well as protein levels (Yao and Rahman, 2012; Lee et al., 2019; Chen et al., 2020). SIRT1 binds directly to the *Akt1* and *mTOR* promoters to promote their transcription, and increased levels of AKT and mTOR expression promote primordial follicle activation. We conducted a clinical translational potential study and found that short-term SIRT1 agonist treatment activates primordial follicles *in vitro* and these follicles develop normally, both in mice and humans. *In vitro* fertilization experiments in mice showed that the quality of oocytes obtained by this method was normal. These results suggest that SIRT1 may be a key protein regulating primordial follicle activation and has certain clinical value (Zhang et al., 2019). Interestingly, overexpression of *Sirt1* was able to delay ovarian aging, and this effect was the same as that of calorie restriction, Calorie restriction protects fertility in female mice by activating SIRT1 (Long et al., 2019; Zhang et al., 2019).

### TGF-β1

TGFB1 transforming growth factor beta 1 (TGF-β1) is a secreted ligand of the TGF-β superfamily. TGF-β binds to various TGF-β receptors leading to recruitment and activation of SMAD family transcription factors that regulate gene expression. The members of TGF-β superfamily, including TGF-β, GDF9, BMP2, BMP4, BMP5, BMP6, BMP7, BMP15, activins and inhibin are expressed by ovarian somatic cells and oocytes in a developmental stage-related manner and function as intraovarian regulators of folliculogenesis (Lee et al., 2001; Hanrahan et al., 2004; Zhao et al., 2016; Vander Ark et al., 2018). Fetal mouse ovary at embryonic stage 18.5 were cultured with the addition of TGF-β ligand for 5–7 days *in vitro* (Wang W. et al., 2014). The results showed that the primordial follicle reserve was reduced and the primordial follicle activation was inhibited. The opposite result was obtained after incubation with SD208, an inhibitor of TGFβ-R1. Further testing found that TGF-β maintained primordial follicle inventory and primordial follicle dormancy by inhibiting the mTOR signaling pathway. In Wang’s research, TGF-β only affects the mTOR signaling pathway, and has no effect on the PI3K signaling pathway (Wang Z.-P. et al., 2014). Zhang’s research showed that mTOR signaling in precursor granulosa cells initiates and regulates primordial follicle activation, after the mTOR pathway in pre-granulosa cells is activated, PI3K key proteins in oocytes are phosphorylated (Zhang et al., 2014). Combined with the research analysis of Wang’s and Zhang’s, we speculate that long-term addition of TGF-β may maintain primordial follicle pool and primordial follicle activation by regulating the mTOR signaling pathway in oocytes. Interestingly, the 4-day-old mouse ovaries were cultured with TGF-β for 2 h, and the phosphorylation of S6 which is a key downstream of the mTOR pathway was significantly increased, p-AKT was not changed, and SMAD3 nuclear export in pre-granulosa cells was increased, thereby promoting primordial follicle activation (Granados-Aparici et al., 2019). In our study (data not shown), mTOR and PI3K signaling pathways are significantly inhibited after adding SD208 to cultured 2 dpp ovaries of mice for 2 days. TGF-β plays different roles in different stages of follicular development. Long-term upregulation of TGF-β and short-time upregulation of TGF-β may lead to different or even opposite results for primordial follicle development.

### NGF

Neurotrophins are growth factors that promote neuronal and non-neuronal cell survival, proliferation and differentiation (Wang W. et al., 2014; Denk et al., 2017). Nerve growth factor (NGF) is a prototype glycoprotein that belongs to the neurotrophins family. NGF contains two classes of receptors: the high affinity receptor tyrosine kinase A (TrkA) and the low-affinity receptor p75 (Chao and Hempstead, 1995; di Mola et al., 2000). The expression of NGF and its receptors is developmentally regulated during folliculogenesis in different mice ovary (Chaves et al., 2010). The number of primordial follicles was not changed, but the number of primary and secondary follicles was significantly reduced in *Ngf* knockout mice. In the absence of NGF, primordial follicles cannot be activated. It is worth noting that exogenous addition of NGF has no effect on the activation of primordial follicles (Kerr et al., 2009; Dorfman et al., 2014; Dorfman et al., 2014). After the ovaries are mechanically injured, the expression of NGF in the stroma cells near the injury site increases rapidly, NGF induces selective activation of primordial follicles near the injury site, including near the ovulation site through the mTOR signaling pathway (He et al., 2017). However, how the NGF in the stroma cells induces the activation of nearby primordial follicles, as well as the specific signal transduction and molecular mechanisms are still unclear. This is a scientific issue that needs attention in the future.

## EGF

Epidermal growth factor (EGF) encodes a member of the epidermal growth factor superfamily, which acts by binding with high affinity to the cell surface receptor, epidermal growth factor receptor (Schneider and Wolf, 2009). In an *in vitro* ovarian culture model, addition of EGF promotes primordial follicle activation by activating the activity of the PI3K pathway in oocytes, and short-term treatment (30 min) can induce the activation of primordial follicles in humans and mice *in vitro*. EGF is a highly effective drug target for primordial follicle activation *in vitro* (Fujihara et al., 2014; Zhang et al., 2020). EGF is highly expressed in zebrafish ovary and testis, EGFRa is expressed in various organs, including the brain, and EGFRb is mainly expressed in the lung and ovary. It is worth noting that only deletion of EGFRa inhibited primordial follicle activation *in vivo*, whereas primordial follicle activation was not affected by deletion of EGF (Song et al., 2022). This suggests that other growth factors may promote primordial follicle activation through EGFR.

### p27

Cyclin-dependent kinase inhibitor 1B (Cdkn1b), also known as p27 or p27Kip1, is a suppressor of cell cycle (Polyak et al., 1994; Chu et al., 2008; Razavipour et al., 2020). The expression of p27 in the ovary during primordial follicle formation and activation is interesting. During primordial follicle formation, p27 is only expressed in the nucleus of somatic cells and not in oocytes. After primordial follicle formation, p27 is expressed in both pre-granulosa cells and oocytes. The expression of p27 is decreased in granulosa cells during primordial follicle activation. In *p27* knockout mice, primordial follicles are formed in advance, and the formed primordial follicles are then activated in advance. In addition, a large number of follicles are atresia and eventually lead to premature ovarian failure. In many studies, it was found that PI3K can regulate the expression of p27, but it is interesting that p27 and PI3K are independent in the process of primordial follicle activation (Rajareddy et al., 2007). However, during chemotherapy, dormant primordial follicles are simultaneously overactivated in the ovary *via* the PI3K/FOXO3a/p27 pathway. Further studies found that in the model of premature ovarian failure induced by cisplatin injection, FOXO3a binds to the promoter of p27 to inhibit its transcription, resulting in excessive activation of primordial follicles. When melatonin and gastrin were injected at the same time, the binding activity of FOXO3a and p27 increased, which promoted the transcription of p27 and saved the over-activation of primitive follicles caused by cisplatin (Jang et al., 2017).

## Clinical application of primordial follicle activation *in vitro*

Primordial follicles (about 1,000) remain in the ovaries of patients with premature ovarian failure, but these primordial follicles are dormant, and their development is not regulated by gonadotropins (Nelson, 2009; De Vos et al., 2010). To utilize the primordial follicle resources in the ovarian tissue of POF patients, the dormant primordial follicles in the ovary must first be activated to develop to a stage when they can respond to gonadotropins, and then use assisted reproductive technology to achieve pregnancy (Telfer and Anderson, 2021). Primordial follicle activation *in vitro* (IVA) was recently developed based on the mechanism of primordial follicle activation, which can help patients with premature ovarian failure to achieve fertility (Yin et al., 2016). In addition, IVA can also be used in middle-aged women who are infertile or infertile due to treatment, allowing them to use their own oocytes to carry on offspring (Bertoldo et al., 2018).

The HIPPO signaling pathway determines organ size and is conserved from *drosophila* to mammals (Seo and Kim, 2018; Ma et al., 2019; Wu and Guan, 2021). Following ovarian damage, disruption of the HIPPO pathway accelerates follicle development, including primordial and growing follicles, which results in increased mice ovarian size. Using the feature of HIPPO signaling pathway can promote primordial follicle activation, combined with agonists of the PI3K or mTOR signaling pathway. A method of primordial follicle activation *in vitro* was developed to help patients with premature ovarian failure successfully have healthy babies (Kawamura et al., 2013; Zhai et al., 2016; Fabregues et al., 2018; Grosbois and Demeestere, 2018; Lee and Chang, 2019; De Roo et al., 2020; Devenutto et al., 2020; Hsueh and Kawamura, 2020; Tanaka et al., 2020; Zhang et al., 2021a). At the same time, factors such as long *in vitro* processing time, poor *in vitro* activation efficiency of primordial follicles, and ethical issues have hindered the clinical application of this technology. Several studies are devoted to improving these adverse factors. The combined use of PI3K and mTOR agonists, resveratrol (SIRT1 agonists), and Rac/Cdc42 activator II (CDC42 agonists) can induce mice ovary primordial follicle activation *in vitro*, which greatly shortens the time of *in vitro* activation. These drugs may be a potential new *in vitro* activation target drugs (Sun et al., 2015; Yan et al., 2018; Zhang et al., 2019). Further research found that orthotopic injection of CDC42 agonist into the ovary can promote the activation of primordial follicles in premature ovarian failure mice and induce human ovary primordial follicle activation *in vitro*. This method by inducing activation directly *in vivo* avoids the unknown risks associated with *in vitro* exposure of ovarian tissue (Zhang et al., 2020). Primordial follicle activation *in vitro*, this new assisted reproductive technology, has been developed to provide new fertility hope for patients with premature ovarian failure.

## Conclusion

**FIGURE 3:**
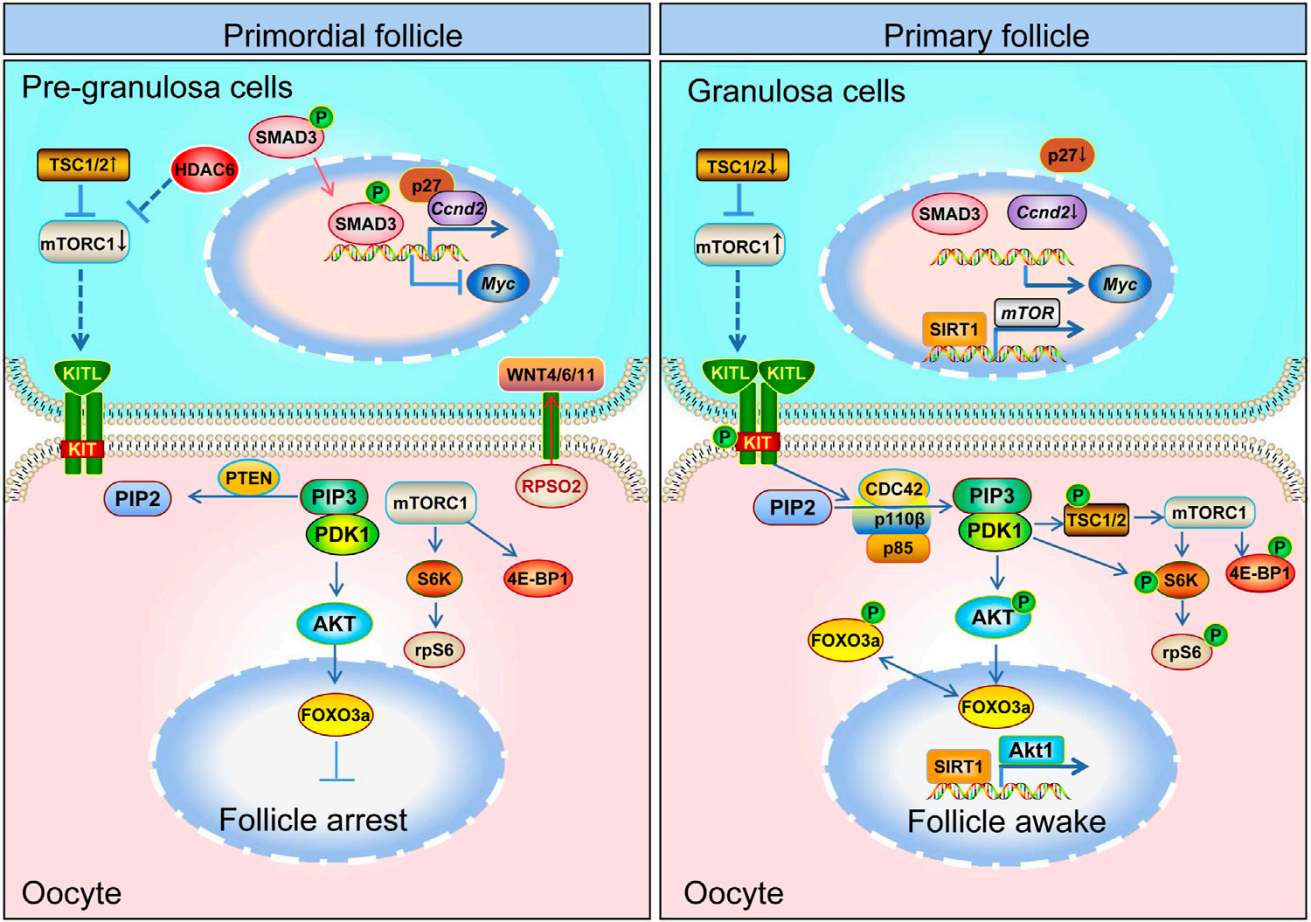
Schematic model depicting the mechanisms of primordial follicle activation. Primordial follicle activation is a result of the delicate interaction of pre-granulosa cells and oocytes and the follicular microenvironment. The mTOR and WNT pathways in pre-granulosa cells, the mTOR and PI3K pathway in oocytes, and the communication channel (KITL-KIT) between pre-granulosa cells and oocytes are all necessary for primordial follicle activation. The mTOR signaling pathway in pre-granulosa cells initiates and regulates primordial follicle activation in adult ovary. The mTOR signal in the pre-granulosa cells senses changes in surrounding nutrients, pressure, *etc.*, so that the pre-granulosa cells secrete more KITL. After KITL binds to the receptor on the oocyte membrane, it activates the PI3K signaling pathway in the oocyte, and then promotes the primordial follicle activation.

The rate of primordial follicle activation controls the length of female fertility. The activation of primordial follicles is mutually regulated by various signaling pathways between oocytes and granulosa cells, and is the result of a close interaction between molecules and between cells (Figure 3). The current study shows that the first wave of primordial follicle activation is determined by PI3K signaling in the oocyte and contributes to female puberty. The second wave primordial follicle activation is determined by mTOR signaling in pre-granulosa cells and determines female fertility throughout life. Understanding the mechanism of primordial follicle activation will help us to further analyze the truth of follicle development and promote the progress of *in vitro* activation technology.
